# The Epidemiology of Osteomyelitis in Children

**DOI:** 10.3390/children8111000

**Published:** 2021-11-03

**Authors:** Nike Walter, Susanne Bärtl, Volker Alt, Markus Rupp

**Affiliations:** 1 Department for Trauma Surgery, University Hospital, 93053 Regensburg, Germany; nike.walter@ukr.de (N.W.); susanne.baertl@ukr.de (S.B.); volker.alt@ukr.de (V.A.); 2 Department for Psychosomatic Medicine, University Hospital, 93053 Regensburg, Germany

**Keywords:** osteomyelitis, bone infection, pediatrics, epidemiology

## Abstract

Pediatric osteomyelitis remains challenging to treat. Detailed epidemiological data are required to estimate future developments. Therefore, we aimed to analyze how the incidence has changed over the last decade depending on age, gender, osteomyelitis subtype, and anatomical localization. Cases were quantified for patients aged 20 years or younger, using yearly reported ICD-10 diagnosis codes from German medical institutions for the time period 2009 to 2019. Incidence rates of osteomyelitis increased by 11.7% from 8.2 cases per 100,000 children in 2009 to 9.2 cases per 100,000 children in 2019. The age-specific incidence rate revealed the highest occurrence of osteomyelitis in patients aged 10–15 years (15.3/100,000 children), which increased by 23% over the observation period, followed by the age group 5–10 years (9.7/100,000 children). In 2019, out of all diagnoses, 39.2% were classified as acute, 38.4% as chronic, and 22.4% were unspecified, whereby chronic cases increased by 38.7%. The lower extremity was mainly affected, with 58.9% of osteomyelitis diagnoses in 2019. In conclusion, pediatric osteomyelitis is a serious issue, even in a developed and industrialized country such as Germany. Considering the recent incidence increase, the permanent need for appropriate treatment should let pediatricians and orthopedic surgeons deal with diagnosis and treatment protocols.

## 1. Introduction

Osteomyelitis is one of the most prevalent musculoskeletal infections in children. Bone infection is most commonly caused by *Staphylococcus aureus* and can occur as a hematogenous infection or after a direct inoculation of bacteria, for instance, due to surgery or open fractures [1,2]. Clinically, the management is challenging. Antibiotic treatment without surgical intervention is a feasible option in cases of acute osteomyelitis, which is regarded within 2 weeks after onset of symptoms. In chronic osteomyelitis, which is defined after more than 2 weeks after onset of symptoms, surgical treatments (including debridement and dead space management) are necessary to achieve eradication of infection [1]. Reliable data on the incidence of pediatric osteomyelitis is of great importance for the evaluation of advances in treatment approaches, prevention strategies, and future developments.

Recently, the epidemiology of osteomyelitis was described for the adult population in Germany, based on nationwide registry data. The authors reported an increase of 10.44% over the last decade and an incidence of 16.7 cases per 100,000 inhabitants for the year 2018 [3]. Some literature also provided insights in the epidemiology of osteomyelitis in pediatric patients [4,5]. However, no current data is available on the epidemiology of acute and chronic osteomyelitis for children in a European country.

Therefore, we analyzed nationwide registry data from Germany, the European country with the highest population numbers. Furthermore, the purpose of this study was to determine changes of pediatric osteomyelitis incidence rates between 2009 and 2019, depending on osteomyelitis subtype, anatomical region, age, and gender.

## 2. Materials and Methods

Data consisting of ICD-10 diagnosis codes, reported annually from German medical institutions, was provided by the Federal Statistical Office of Germany (Destatis) for the time period 2009 to 2019. These include all inpatient diagnoses, which are reported from medical institutions of all 16 German federal states, including private ones. Patients aged 20 years or younger diagnosed with osteomyelitis were identified using the ICD-10 code “M86.-”. The prevalence was estimated for gender, age groups in five-year increments, osteomyelitis subtype, and affected region, respectively. The subgroup “acute osteomyelitis” was composed by applying the ICD-10 codes “M86.0, M86.1, M86.2”; whereas, chronic osteomyelitis cases were estimated using the codes “M86.3, M86.4, M86.5, M86.6”. Unspecified osteomyelitis cases were determined by “M86.8, M86.9”. For the upper extremity, the subgroup the codes “-1, shoulder”, “-2, humerus”, “-3, radius and ulna”, and “-4, hand” were included. The lower extremity subgroup was compiled using the codes “-5, femur”, “-6, tibia and fibula” and “-7, ankle and foot”. The incidence rate was estimated based on Germany’s total population of those aged 20 years or younger for each year provided by Destatis [6]. Data were analyzed with SPSS Version 26.0 (IBM, SPSS Inc., Armonk, NY, USA).

## 3. Results

**Figure 1:**
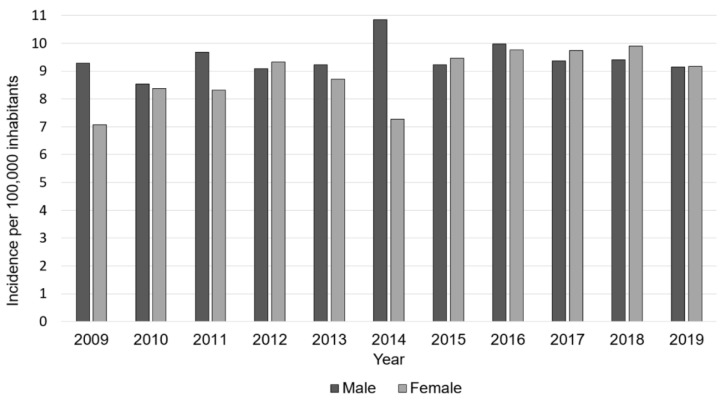
Development of osteomyelitis incidence from 2009 to 2019, divided by gender. The light grey bars present the incidence of male cases, and the dark grey bars present the incidence of female cases.

In 2019, 1486 osteomyelitis cases in the German population aged younger than 20 years were registered. Compared to 1336 cases in 2009, the incidence increased by 11.7%, from 8.2 cases per 100,000 inhabitants to 9.2 cases per 100,000 inhabitants. Since 2009, total numbers steadily increased, reaching a maximum in 2016, followed by a subsequent decrease of diagnoses. Over the years, male cases were slightly more often reported than female cases; whereas, the gender distribution was equal in 2019 (Table 1, Figure 1).

**Figure 2:**
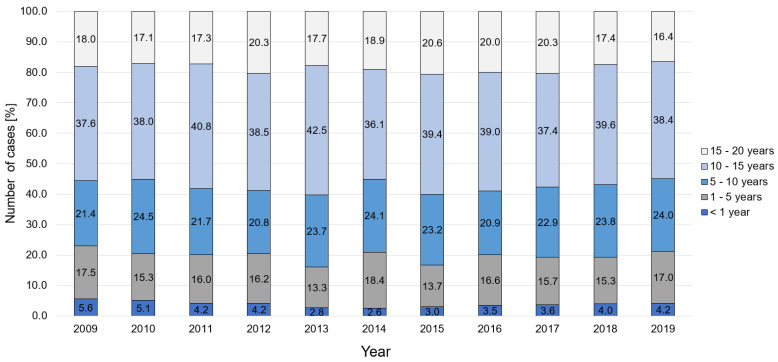
Age-dependent changes in incidence of osteomyelitis cases.

Regarding the internal infection proportion, most patients were aged 10–15 years (38.4%), followed by patients aged 5–10 years (24.0%) and patients aged 1–5 years (17.0%) in 2019 (Figure 2). The age-specific incidence rate per 100,000 children revealed the highest occurrence of osteomyelitis in patients aged 10–15 years (15.3/100,000 children), which increased by 23% over the observation period. The second most affected group were children aged 5–10 years, with an incidence rate of 9.7/100,000 children; whereby, cases increased by 25.3% in the last decade. This was followed by an incidence of 8.1/100,000 children aged younger than 1 year; whereby, less children were diagnosed with osteomyelitis in comparison to 2009 (−25.9%). Additionally, for children aged 1–5 years, the incidence decreased by 5.6%, to 6.4 cases/100,000 children (Table 2).

**Figure 3:**
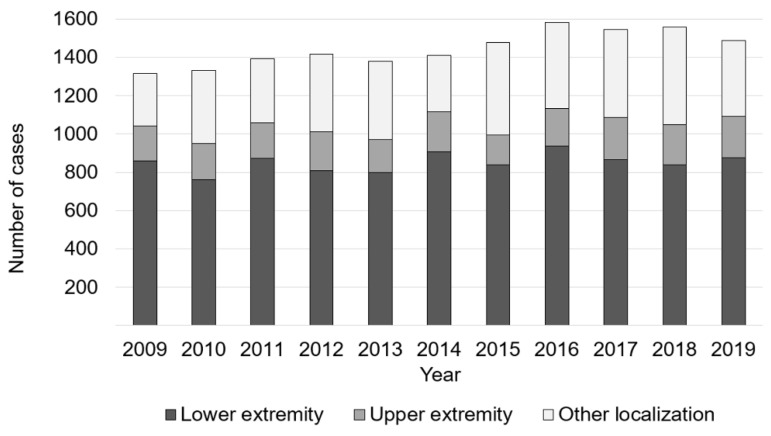
Anatomical region dependent changes osteomyelitis cases.

The lower extremity was the most frequently infected region, with 58.9% in 2019. Here, the number of cases increased from 816 in 2009 to 875 in 2019. In 14.7% of all cases bone, infection was present at the upper extremity. Other anatomical localizations were affected in 26.4% (Figure 3).

**Figure 4:**
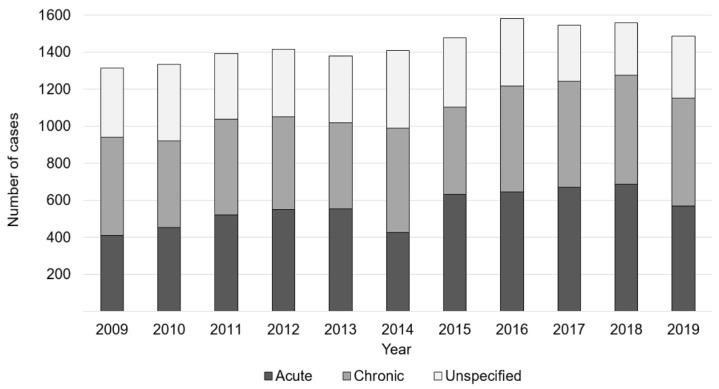
Development of osteomyelitis prevalence, classified as acute, chronic, and unspecified.

In 2019, out of all diagnoses, 39.2% were classified as acute, 38.4% as chronic, and 22.4% were unspecified. Cases which were classified as acute increased by 10.2%, from 529 to 583 between 2009 and 2019. Additionally, chronic osteomyelitis cases rose by 38.7%, from 411 to 570 cases; whereas, unspecified osteomyelitis diagnoses declined by 11.0%, from 374 to 333 (Figure 4).

## 4. Discussion

In this cross-sectional study, developments of the epidemiology of pediatric osteomyelitis were analyzed for osteomyelitis subtype, anatomical localization, gender, and age group, respectively. The results show that the annual incidence increased by 11.7%, to 9.2 cases per 100,000 children. The incidence is comparable to other studies. For instance, the incidence rate of non-vertebral osteomyelitis was calculated as 10/100,000 children in Norway [4] and 7.8–9.1/100,000 children in South Korea [7]. However, country-specific differences are also evident. A ten year retrospective review of pediatric osteomyelitis treatment in two orthopedic departments in New Zealand reported an annual rate of 1:4000 for acute hematogenous in patients aged 0–15; whereby, the incidence declined over the years [8]. In the U.S., hospitalization rates ranged from 1.34 to 1.66/100,000 children for acute osteomyelitis, which is less than estimated for Germany [5]. Among the countries, osteomyelitis incidence in indigenous children of northern Australia was the highest reported globally, with rates of 82 per 100,000 children, compared to 9 per 100,000 for nonindigenous children [9]. Where some authors report decreases in incidence [10], others report an increasing incidence [11], which could partly reflect advances in microbiological diagnosis [12,13]. Here, the age-specific incidence rates of osteomyelitis showed decreasing trends among children aged <1 year; whereas, the incidence rates increased among children aged >5 years. This was in contrast to other studies, reporting more cases in children aged <3 years, compared with older children under the age of 16 years (OR 2.9, 95%: CI 2.3–3.7) [4]. The study further shows that gender distribution was equal, which is consistent with findings from studies performed in countries such as Norway [4]. However, other publications reported more frequent osteomyelitis diagnoses in males than in females [11,14]. In the reported data, the lower extremity was more often affected, which is consistent with the literature, reporting the highest infection rates for the femur and tibia [15]. In addition, out of all diagnoses, 39.2% were classified as acute, 38.4% as chronic, and 22.4% were unspecified in 2019. The widely used classification by Waldvogel and colleagues distinguishes between acute and chronic; whereby, acute osteomyelitis is defined by symptoms for a duration of less than two weeks [1]. However, different definitions exist in the literature [16], which might influence the presented statistics and results in potential misclassification of acute and chronic diagnoses. Furthermore, chronic cases heightened by 38.7% over the years. Hence, early diagnosis, advances in prevention strategies, and prompt treatment are of fundamental importance, as a mature biofilm limits the susceptibility to antibiotics, leading to a requirement of surgical treatment [17].

The biggest limitation of this study is due to the fact that the analysis was solely based on inpatient data. However, surgical treatment is required in the majority of osteomyelitis cases. Furthermore, the analysis of ICD-10 codes does not allow to distinguish between hematogenous and postoperative infections, or to determine possible driving comorbidity factors, such as immune deficiencies or fractures. In the same stance, it was not possible to derive information about the underlying pathogens and treatment procedures. Finally, correct coding cannot be assured.

## 5. Conclusions

In conclusion, pediatric osteomyelitis remains a challenge even in a developed and industrialized country such as Germany. Considering the recent incidence increase, the permanent need for appropriate treatment should let pediatricians and orthopedic surgeons deal with diagnosis and treatment protocols, to maintain and further improve the treatment for our young patients.

## Figures and Tables

**Table 1:** Pediatric osteomyelitis incidence changes between 2009 and 2019.

Year	Total Numbers	Incidence per 100,000 Children	Relative to 2009 [%]	Ratio Male/Female [%]
2009	1336	8.2		53/47
2010	1314	8.5	+3.0	58/42
2011	1333	9.0	+9.8	52/48
2012	1417	9.2	+12.1	55/45
2013	1380	9.0	+9.4	51/49
2014	1409	9.1	+10.99	53/47
2015	1475	9.3	+13.8	61/39
2016	1581	9.9	+20.3	51/49
2017	1545	9.6	+16.4	52/48
2018	1560	9.6	+17.4	51/49
2019	1486	9.2	+11.7	50/50

**Table 2:** Historic development of age-specific incidence rate, per 100,000 children.

Year	<1 Year Old	1–5 Years Old	5–10 Years Old	10–15 Years Old	15–20 Years Old
2009	11.0	6.7	7.7	12.5	5.5
2010	10.0	6.0	9.2	12.8	5.5
2011	8.8	6.6	8.7	14.0	6.0
2012	8.9	6.8	8.5	14.3	7.1
2013	5.6	5.4	9.4	15.7	6.0
2014	5.0	7.4	9.7	13.7	6.6
2015	6.0	5.6	9.6	15.8	7.3
2016	7.1	7.0	9.2	16.7	7.6
2017	7.1	6.4	9.7	15.6	7.4
2018	7.9	6.1	10.1	16.6	6.5
2019	8.1	6.4	9.7	15.3	6.0

## Data Availability

The data that support the findings of this study are available on request from the corresponding author.
